# Metagenomic Analysis of the Abundance and Composition of Antibiotic Resistance Genes in Hospital Wastewater in Benin, Burkina Faso, and Finland

**DOI:** 10.1128/msphere.00538-22

**Published:** 2023-02-02

**Authors:** Melina A. Markkanen, Kaisa Haukka, Katariina M. M. Pärnänen, Victorien Tamegnon Dougnon, Isidore Juste O. Bonkoungou, Zakaria Garba, Halidou Tinto, Anniina Sarekoski, Antti Karkman, Anu Kantele, Marko P. J. Virta

**Affiliations:** a Department of Microbiology, University of Helsinki, Helsinki, Finland; b Multidisciplinary Center of Excellence in Antimicrobial Resistance Research, University of Helsinki, Helsinki, Finland; c Research Unit in Applied Microbiology and Pharmacology of Natural Substances, Polytechnic School of Abomey-Calavi, University of Abomey-Calavi, Abomey-Calavi, Benin; d Department of Biochemistry and Microbiology, University Joseph Ki-Zerbo, Ouagadougou, Burkina Faso; e Clinical Research Unit Nanoro, Institute for Research in Health Sciences, National Center for Scientific and Technological Research, Ouagadougou, Burkina Faso; f Meilahti Vaccine Research Center MeVac, Department of Infectious Diseases, University of Helsinki and Helsinki University Hospital, Helsinki, Finland; g Human Microbiome Research Program, Faculty of Medicine, University of Helsinki, Helsinki, Finland; JMI Laboratories

**Keywords:** antibiotic resistance, West Africa, hospital wastewater (HWW), carbapenemase, colistin resistance, metagenomes

## Abstract

Antibiotic resistance is a global threat to human health, with the most severe effect in low- and middle-income countries. We explored the presence of antibiotic resistance genes (ARGs) in the hospital wastewater (HWW) of nine hospitals in Benin and Burkina Faso, two low-income countries in West Africa, with shotgun metagenomic sequencing. For comparison, we also studied six hospitals in Finland. The highest sum of the relative abundance of ARGs in the 68 HWW samples was detected in Benin and the lowest in Finland. HWW resistomes and mobilomes in Benin and Burkina Faso resembled each other more than those in Finland. Many carbapenemase genes were detected at various abundances, especially in HWW from Burkina Faso and Finland. The *bla*_GES_ genes, the most widespread carbapenemase gene in the Beninese HWW, were also found in water intended for hand washing and in a puddle at a hospital yard in Benin. *mcr* genes were detected in the HWW of all three countries, with *mcr-5* being the most common *mcr* gene. These and other *mcr* genes were observed in very high relative abundances, even in treated wastewater in Burkina Faso and a street gutter in Benin. The results highlight the importance of wastewater treatment, with particular attention to HWW.

**IMPORTANCE** The global emergence and increased spread of antibiotic resistance threaten the effectiveness of antibiotics and, thus, the health of the entire population. Therefore, understanding the resistomes in different geographical locations is crucial in the global fight against the antibiotic resistance crisis. However, this information is scarce in many low- and middle-income countries (LMICs), such as those in West Africa. In this study, we describe the resistomes of hospital wastewater in Benin and Burkina Faso and, as a comparison, Finland. Our results help to understand the hitherto unrevealed resistance in Beninese and Burkinabe hospitals. Furthermore, the results emphasize the importance of wastewater management infrastructure design to minimize exposure events between humans, HWW, and the environment, preventing the circulation of resistant bacteria and ARGs between humans (hospitals and community) and the environment.

## INTRODUCTION

The global antibiotic resistance crisis has multifaceted effects on human and animal health and comes with substantial economic losses (1). Due to limitations in diagnostic testing in low-resource settings in low- and middle-income countries (LMICs), broad-spectrum antibiotics are often used empirically without microbiological verification of the causative pathogen or its sensitivity to different antibiotics (2). In addition, unregulated access to antibiotics results in self-medication for humans and animals in these countries (3–5).

Acquired, potentially mobile antibiotic resistance genes (ARGs) have a pronounced clinical relevance and impact on the current antimicrobial resistance (AMR) problem (6, 7). Furthermore, the genetic context of the ARG (e.g., whether under a strong promoter or not) influences its expression and the resulting resistance phenotype (8). Class 1 integrons are strongly linked to the dissemination of clinically relevant acquired ARGs (9, 10). Although integrons are not mobile as such, multidrug resistance gene cassettes carried by integrons can be transferred to new hosts: for example, via plasmids (11). The *intI1* and *qacE*Δ genes (genes encoding the integron integrase and quaternary ammonium compound resistance) are typically used as markers for class 1 integrons (10, 12).

The use of broad-spectrum antibiotics has increased in clinical practice as a consequence of the increased prevalence of extended-spectrum β-lactamase-producing *Enterobacteriaceae* (ESBL-PE) (13–15). The carbapenem resistance-encoding genes *bla*_GES_, *bla*_IMP_, *bla*_KPC_, *bla*_NDM_, *bla*_OXA-48_, *bla*_OXA-58_, and *bla*_VIM_, which are typically carried by plasmids, have emerged and spread around the world during the past 3 decades (16–19). Colistin is a last-resort antibiotic used for treating infections caused by multidrug-resistant and extensively drug-resistant bacteria, such as those resistant to carbapenems (20). The rapid emergence of colistin resistance mediated by *mcr* genes threatens the efficacy of colistin in clinical use (21, 22).

Although AMR is a global concern, the crisis dramatically affects LMICs, such as those in West Africa (2, 23–26). Lack of research data is one major factor hindering tackling the AMR problem in these countries (2, 23, 27, 28). Despite these gaps in resistance surveillance data, it is well known that the level of AMR is elevated in LMICs, including African countries (14, 24, 29, 30). In contrast, in Northern European countries, such as Finland, AMR occurrence is among the lowest globally, both in the community (30, 31) and in health care settings (32).

Hospital wastewater (HWW) from health care facilities is at the frontline of AMR emergence and spread due to the frequent use of antibiotics and the presence of immunocompromised patients (33). Thus, we set out to investigate the AMR situation and the characteristics of the resistomes in nine hospitals in two West African countries, Benin and Burkina Faso, where prior data were scarce (14). For comparison, we analyzed samples from six hospitals in Finland, where the level of AMR was expected to be low (32). We used shotgun metagenomic sequencing to obtain a holistic view of the resistomes, mobilomes (a set of ARGs and mobile genetic elements [MGEs], respectively), and microbial communities present in the studied environments.

## RESULTS

### General features of resistomes, mobilomes, and taxonomical compositions in HWW and other water samples from Benin, Burkina Faso, and Finland.

**FIG 1:**
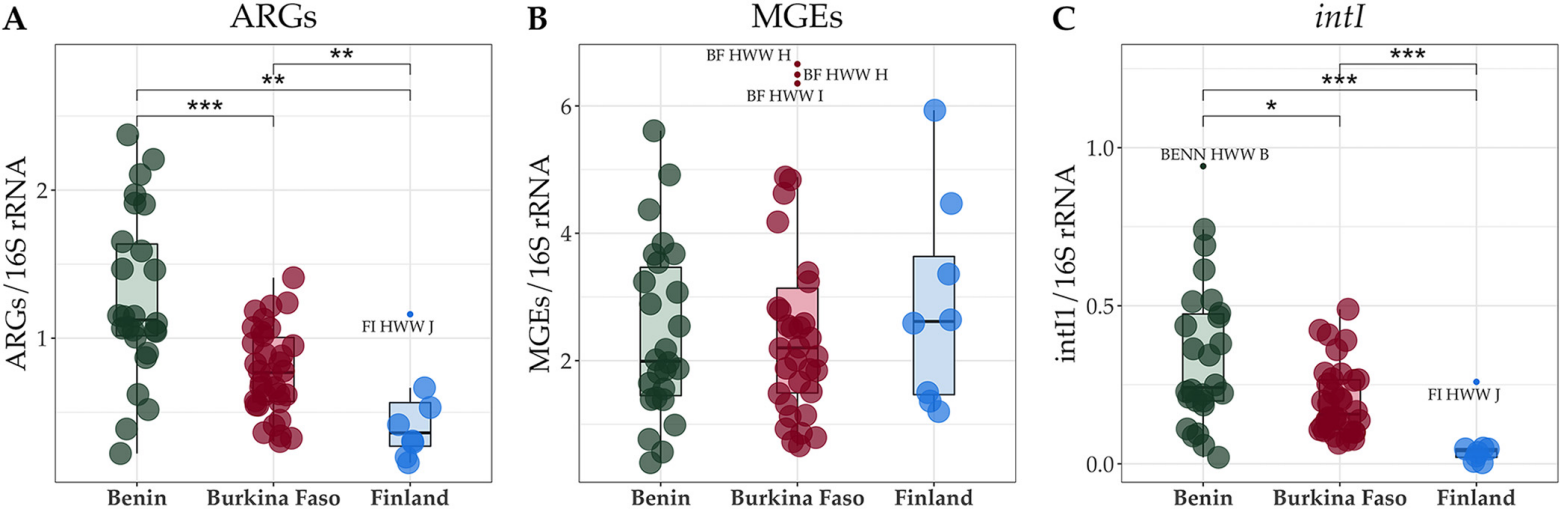
Sum of the relative abundances of (A) ARGs, (B) MGEs, and (C) class 1 integrons (*intiI1*) in HWW samples from Benin, Burkina Faso, and Finland. The gene counts were normalized to 16S rRNA gene counts and gene lengths. Country medians are shown as horizontal lines, and the interquartile ranges (25th and 75th quartiles) as box plot hinges. The horizontal lines represent the highest and lowest values. Outliers are defined as values higher or lower than 1.5 times the upper or lower quartiles, respectively, and denoted with a text label referring to the category represented by that sample. The box plots were drawn after excluding the outliers. The comparisons between countries were computed using the pairwise Wilcoxon rank sum test, where the *P* values were adjusted for multiple testing using the Benjamini-Hochberg algorithm. The significance levels are as follows: *, *P* ≤ 0.05; **, *P* ≤ 0.01; ***, *P* ≤ 0.001.

We studied hospital wastewater (HWW) collected from Benin, Burkina Faso, and Finland (Table 1; see Data Set S1, Sheets 1 and 2, in the supplemental material; see Fig. S1 and S2 in the Supplemental Data Repository, https://data.mendeley.com/datasets/9wxb37t49z/1). In addition, 11 non-HWW water samples, such as those collected from rivers and near or within the hospital environment, were analyzed to obtain a more comprehensive understanding of the ARG prevalence in Benin and Burkina Faso. On average, 31 million sequence reads per sample were analyzed. HWW from Benin showed the highest and HWW from Burkina Faso the second-highest abundance of all detected ARGs normalized to bacterial 16S rRNA genes (the sum of the relative abundance of ARGs). On the other hand, the sum of the relative abundance of ARGs was the lowest in HWW from Finland (Fig. 1A). Also, the lowest diversity of ARGs was observed in HWW from Finland and the highest in Burkina Faso (see Fig. S3A in the Supplemental Data Repository).

**TABLE 1:** Sample information

Sample type and ID^*a*^	Country	Category	*N*	Hospital/area	Collection date (day/mo/yr)	HWW
HWW hospital samples						
BH01–BH09	Benin	BENN HWW A	7	A	27/11/19	Yes
BH27–BH39		BENN HWW B	11	B	29/11/19	Yes
BH44–BH50		BENN HWW C	5	C	9/12/19	Yes
BH58–BH61		BENN HWW D	3	D	11/12/19	Yes
BFH1–BFH4	Burkina Faso	BF HWW E	2	E	22/11/19	Yes
BFH6–BFH12		BF HWW F	6	F	28/11/19	Yes
BFH13–BFH15		BF HWW G	3	G	28/11/19	Yes
BFH16–BFH28		BF HWW H	10	H	4/12/19	Yes
BFH29–BFH41		BF HWW I	13	I	12/12/19	Yes
FH1	Finland	FI HWW J	1	J	20/1/20	Yes
FH2		FI HWW K	1	K	20/1/20	Yes
FH3		FI HWW L	1	L	20/1/20	Yes
FH4–FH6		FI HWW M	3	M	20/1/20	Yes
FH7		FI HWW N	1	N	23/1/20	Yes
FH9		FI HWW O	1	O	28/1/20	Yes
Other samples						
BH11	Benin	BENN well water A (drinking)	1	A	27/11/19	No
BH13		BENN street gutter B	1	B	27/11/19	No
BH48		BENN puddle at yard C	1	C	9/12/19	No
BH52		BENN hand-washing C	1	C	9/12/19	No
BSE100		BENN river P (drinking)	1	P	4/12/19	No
BSE74		BENN river Q (drinking)	1	Q	4/12/19	No
BSE79		BENN river R (drinking)	1	R	4/12/19	No
BSE93		BENN tap water S (drinking)	1	S	4/12/19	No
BFH26	Burkina Faso	BF exit after biological treatment	1	H	4/12/19	No
BFH27		BF wetland receiving treated HWW	1	H	4/12/19	No
BFH42		BF receiving river after WWTP	1	T	12/12/19	No

a HWW samples from hospitals in Benin (*n* = 26), Burkina Faso (*n* = 34), and Finland (*n* = 8) are denoted at the top, while the other samples are noted at the bottom.

10.1128/msphere.00538-22.5 DATA SET S1 The metadata of the samples investigated in this study are shown in Sheet 1, and those for all samples included in this study are shown in Sheet 2. The table columns on both sheets describe the samples by different variables. These include, for example, hospital code, type of hospital, and the explanation of the sample collection site. Download Data Set S1, XLSX file, 0.1 MB. Copyright © 2023 Markkanen et al. 2023 Markkanen et al. https://creativecommons.org/licenses/by/4.0/ This content is distributed under the terms of the Creative Commons Attribution 4.0 International license.

According to the Kruskal-Wallis test, there were significant (*P* < 0.005) country-wise differences in the sums of relative abundances of ARGs (Fig. 1A) and class 1 integron genes (*intI1*) (Fig. 1C), but not when no particular type of MGE was specified (Fig. 1B). The significant differences were investigated further using the Wilcoxon rank sum test, where the *P* values were adjusted for multiple testing using the Benjamini-Hochberg algorithm. The differences in *intI1* followed a similar pattern in country-wise comparisons to ARGs (Fig. 1C). Contigs where multiple ARGs, such as carbapenemase or ESBL variants of *bla*_GES_ and quinolone resistance genes (*qnrVC*), were located in proximity to each other were identified (Fig. S4 in the Supplemental Data Repository). These contigs might indicate gene cassettes carried by class 1 integron elements as previously reported for *bla*_GES_ and *qnrVC* in various ARG combinations (8, 34, 35). In contrast to *intI1* and *qacE*Δ, no significant correlations were observed between ARGs and *int2* or *int3* (Fig. S5C and D in the Supplemental Data Repository), except with HWW from Burkina Faso (Fig. S5D in the Supplemental Data Repository).

**FIG 2:**
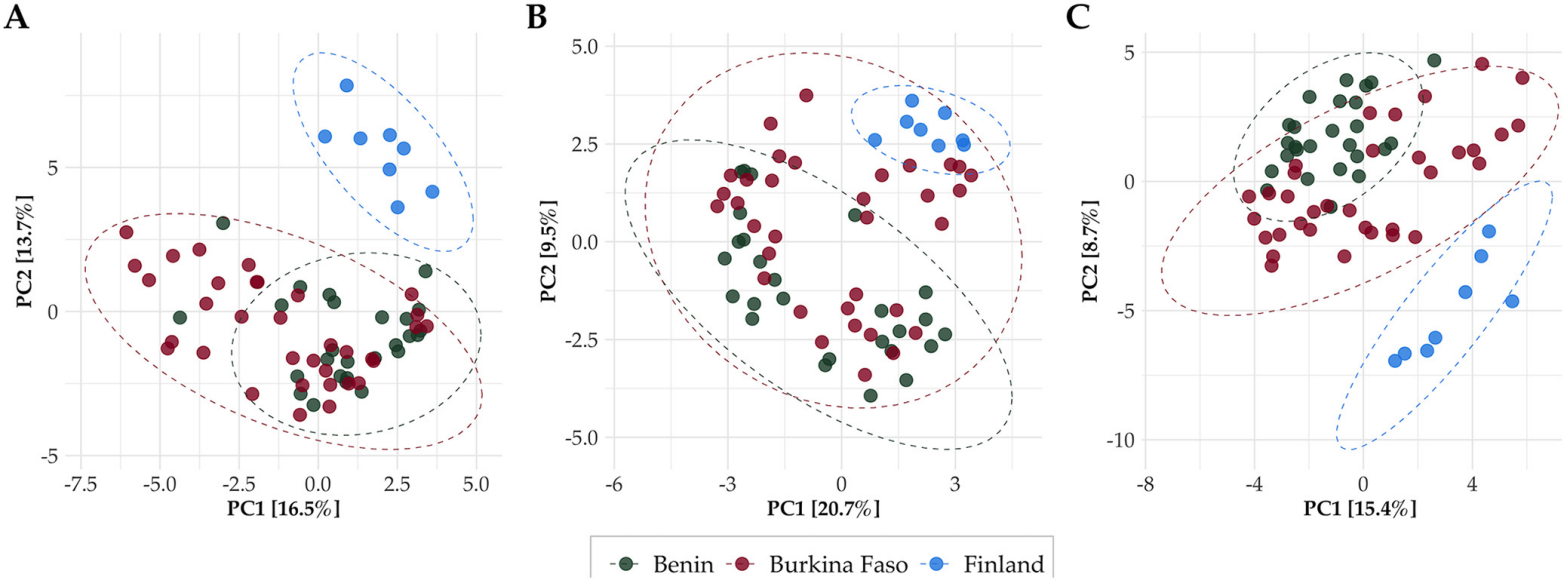
Principal-component analysis (PCA) showing the significant dissimilarities of (A) resistomes, (B) taxonomical composition Metaphlan3, and (C) mobilomes in HWW from Benin, Burkina Faso, and Finland. Count data were transformed using centered log ratio transformation (clr). Confidence ellipses are drawn for visualization and represent 95% confidence levels.

Compositional data analysis (CoDa) methods were applied to investigate the ordination of the samples from the different countries by their resistome, mobilome, and taxonomical composition with respect to each other. Centered log ratio (clr) transformation, which uses the geometric mean of the sample vector as reference (36), was applied to transform the count data for the ordinations. The significance of the distances between samples from country pairs was calculated on untransformed count data using Aitchison distance, which corresponds to Euclidean distances between clr-transformed sample abundance vectors (37). HWW resistomes from Benin and Burkina Faso resembled each other and formed clusters distinct from the resistomes from Finland (permutational multivariate analysis of variance [PERMANOVA]; *P* < 0.001; Benin versus Finland [BENN-FI], *R*^2^ = 0.211, Burkina Faso versus Finland [BF-FI]; *R*^2^ = 0.160; Benin versus Burkina Faso [BENN-BF], *R*^2^ = 0.0801) (Fig. 2A; Table S1A). Country (BENN-FI, BENN-BF, and BF-FI) explained less of the variance between taxonomical compositions than variance between resistomes (PERMANOVA; *P* < 0.001; BENN-FI, *R*^2^ = 0.178; BF-FI, *R*^2^ = 0.104; *P* < 0.005; BENN-BF, *R*^2^ = 0.0441) (Fig. 2B; Table S1B). Similar to resistomes, distinct country-wise clusters were seen in the ordinations of mobilomes (PERMANOVA; *P* < 0.001; BENN-FI, *R*^2^ = 0.196; BF-FI, *R*^2^ = 0.103; *P* < 0.005; BENN-BF, *R*^2^ = 0.0478) (Fig. 2C; Table S1C). When non-HWW water samples were included in the analysis, these samples seemed to be located approximately within the clusters of their respective countries (PERMANOVA; *P* < 0.001) (Table S1D to F; Fig. S6 in the Supplemental Data Repository). Since the HWW resistomes of Benin and Burkina Faso differed notably from the resistomes of Finland, the drivers for these differences were subsequently investigated.

10.1128/msphere.00538-22.1 TABLE S1 PERMANOVA results for cluster analysis of resistomes, mobilomes, and taxonomical compositions of HWW samples (A to C) and all samples (D to F). Download Table S1, XLSX file, 0.02 MB. Copyright © 2023 Markkanen et al. 2023 Markkanen et al. https://creativecommons.org/licenses/by/4.0/ This content is distributed under the terms of the Creative Commons Attribution 4.0 International license.

### Significantly differentially abundant ARGs.

ARGs significantly differentially abundant in HWW from each country-wise comparison were investigated. For that, analysis by the ANOVA-Like Differential Expression tool for high-throughput sequencing data (ALDEx2) was performed with additive log ratio (alr) transformation (36). alr transformation uses a single component (here, the 16S rRNA counts) as the reference for analyzing all individual components.

**FIG 3:**
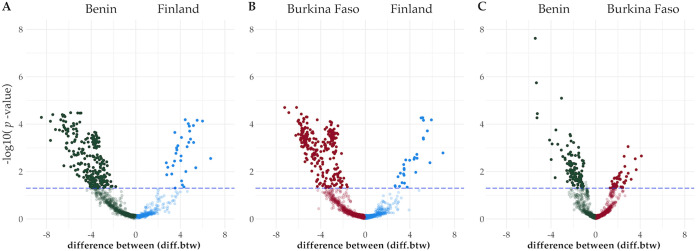
Significantly differentially abundant ARGs in HWW in pairwise comparisons in Benin versus Finland, Burkina Faso versus Finland, and Benin versus Burkina Faso were defined using ALDEx2 (36) with 16S rRNA as the reference gene. The difference-between (diff.btw) values represent the difference in alr-transformed values of each ARG in samples from the compared countries. For instance, in panel A, significantly differentially abundant ARGs for Benin (green dots above the blue dotted line) have negative diff.btw values (*x* axis), while ARGs that were significantly differentially abundant in Finland (blue dots above the blue dotted line) have positive values. The dotted line represents a *P* value of <0.05 of the Wilcoxon rank sum test, where the *P* values were adjusted for multiple testing using the Benjamini-Hochberg algorithm (wi.eBH). These *P* values were log_10_ transformed for the figure.

In the comparisons Benin versus Finland and Burkina Faso versus Finland, ARGs significantly differentially abundant in HWW from Finland were fewer than those from Benin or Burkina Faso (Fig. 3A and B) (BENN [*n* = 258]-FI [*n* = 33]; BF [*n* = 257]-FI [*n* = 29]). In addition, the ARGs that were significantly differentially abundant in HWW from Finland in both comparisons (Benin versus Finland and Burkina Faso versus Finland) were primarily the same (Table 2; Table S1A and B). *bla*_OXA-211_-like genes and *erm*(B) macrolide resistance genes were characteristic of HWW from Finland (Table 2 [and see Table S1A to C for all results]). Those ARGs that were characteristic of HWW from Benin included tetracycline resistance gene *tetA*, quinolone resistance genes *qnrVC*, and ESBL gene *bla*_VEB_ (Table 2; and Table S1A). For Burkina Faso, significantly differentially abundant ARGs included *bla*_CMY_ genes, trimethoprim resistance genes of the gene family *dfrA15*, and genes of *bla*_OXA-10_- and *bla*_OXA-46_-like gene families (Table 2; Table S1B).

**TABLE 2:** Top 10 differentially abundant ARGs in HWW from Benin, Burkina Faso, and Finland^*a*^

Comparison	wi.eBH	Diff.btw	Gene	Gene family
Benin vs Finland				
Benin	0.000032	−5.811741	*tet(A)_1*	*tet*(A)
	0.000033	−5.267743	*tet(A)_6*	*tet*(A)
	0.000034	−4.932008	*tet(A)_4*	*tet*(A)
	0.000034	−4.842077	*tet(A)_5*	*tet*(A)
	0.000037	−7.475163	*qnrVC4_1*	*qnrVC4*
	0.000042	−6.688637	*blaVEB-2_1*	*bla*_VEB_
	0.000043	−6.521372	*blaVEB-6_1*	*bla*_VEB_
	0.000045	−6.600232	*blaVEB-7_1*	*bla*_VEB_
	0.000047	−6.629523	*blaVEB-1_1*	*bla*_VEB_
	0.000058	−8.506581	*lnu(F)_3*	*lnu*(F)
Finland	0.000065	5.930829	*blaOXA-373_1*	*bla*_OXA-211_-like
	0.000068	4.334039	*erm(B)_18*	*erm*(B)
	0.000079	5.493227	*blaOXA-212_1*	*bla*_OXA-211_-like
	0.000085	4.834230	*erm(B)_21*	*erm*(B)
	0.000113	4.735091	*blaOXA-309_1*	*bla*_OXA-211_-like
	0.000118	5.320211	*erm(B)_10*	*erm*(B)
	0.000189	3.499903	*erm(B)_9*	*erm*(B)
	0.000225	5.338762	*blaOXA-334_1*	*bla*_OXA-211_-like
	0.000407	4.719647	*blaOXA-299_1*	*bla*_OXA-299_-like
	0.000409	4.664600	*blaOXA-281_1*	*bla*_OXA-211_-like
Burkina Faso vs Finland				
Burkina Faso	0.000016	−7.153927	*blaCMY-4_1*	*bla*_CMY-150_
	0.000018	−6.185994	*dfrA15_2*	*dfrA15*
	0.000031	−6.923325	*blaOXA-101_1*	*bla*_OXA-10_-like
	0.000039	−5.625515	*dfrA15_4*	*dfrA15*
	0.000047	−5.795837	*blaCMY-2_1*	*bla*_CMY-150_
	0.000051	−5.223876	*blaVEB-6_1*	*bla*_VEB_
	0.000051	−5.261070	*blaOXA-56_1*	*bla*_OXA-10_-like
	0.000066	−4.173189	*tet(A)_1*	*tet*(A)
	0.000068	−5.930935	*blaCMY-121_1*	*bla*_CMY-150_
	0.000072	−5.478843	*blaOXA-46_1*	*bla*_OXA-46_-like
Finland	0.000043	5.262960	*blaOXA-334_1*	*bla*_OXA-211_-like
	0.000055	5.481699	*blaOXA-299_1*	*bla*_OXA-299_-like
	0.000057	5.261032	*blaOXA-309_1*	*bla*_OXA-211_-like
	0.000071	6.062276	*blaOXA-212_1*	*bla*_OXA-211_-like
	0.000229	5.378873	*blaOXA-373_1*	*bla*_OXA-211_-like
	0.000268	5.311696	*blaOXA-281_1*	*bla*_OXA-211_-like
	0.000513	5.474025	*blaOXA-280_1*	*bla*_OXA-211_-like
	0.001580	7.159558	*blaOXA-211_1*	*bla*_OXA-211_-like
	0.001721	4.643516	*erm(B)_10*	*erm*(B)
	0.002353	3.711949	*erm(B)_6*	*erm*(B)
Benin vs Burkina Faso				
Benin	1.64E−08	−5.4719564	*blaOXA-129_1*	*bla*_OXA-5_-like
	1.97E−06	−5.3861927	*aac(6′)-IIc_1_NC*	*aac(6′)-Iic*
	8.19E−06	−3.0755306	*blaOXA-256_1*	*bla*_OXA-10_-like
	4.10E−05	−5.3303508	*aph(2″)-Ib_1*	*aph(2″)-Ib*
	5.43E−05	−5.244107	*aac(6′)-Im_1*	*aac(6′)-Im*
	0.00020678	−3.4867524	*lnu(C)_1*	*lnu*(C)
	0.0002553	−2.2747178	*tet(C)_3*	*tet*(C)
	0.00030175	−2.3241004	*tet(C)_2*	*tet*(C)
	0.00051131	−4.0449182	*aph(2″)-Ib_2*	*aph(2″)-Ib*
	0.00051266	−2.256968	*tet(C)_1_NC*	*tet*(C)
Burkina Faso	0.00091224	2.91935432	*tet(39)_1*	*tet*(39)
	0.00151264	4.1710954	*blaBEL-1_1*	*bla*_BEL_
	0.002193	2.53504117	*cmlB1_1*	*cmlB*
	0.00328808	3.4417663	*blaOXA-58_1*	*bla*_OXA-58_-like
	0.00556017	2.5513906	*dfrB5_1*	*dfrB1*
	0.00999705	2.36292741	*blaCMY-15_1*	*bla*_CMY-150_
	0.01017209	2.18229534	*blaCMY-95_1*	*bla*_CMY-150_
	0.0104837	3.39104568	*blaBEL-3_1*	*bla*_BEL_
	0.01139229	2.93238914	*blaOXA-397_1*	*bla*_OXA-58_-like
	0.01498384	1.9958258	*blaCMY-94_1*	*bla*_CMY-150_

a The diff.btw values represent the median difference in the alr-transformed count data between the compared HWW. The *P* values of the Wilcoxon rank sum test, where the *P* values were adjusted for multiple testing using the Benjamini-Hochberg algorithm, are indicated in the table as “wi.eBH.” The table was sorted by the wi.eBH value before subdividing the entries into the top 10 differentially abundant ARGs by country: Benin, Burkina Faso, or Finland. The ARGs were clustered into gene families (Gene family column) based on 90% shared sequence identity using CD-HIT (80). In the case of *bla*_OXA_ genes, the gene family naming followed the scheme by Naas and colleagues (46).

The comparison between HWW from Benin and Burkina Faso revealed fewer differentially abundant ARGs between these countries (BENN [*n* = 118]-BF [*n* = 53]) than in the comparisons with Finnish HWW. In addition, the volcano plot in Fig. 3C is more skewed toward the center (diff.btw value of zero), thus referring to less drastic differences in this comparison. These results support the previous notion that the resistomes in HWW from Benin and Burkina Faso were more similar to each other than those from Finland (Table 2; Table S1A to C). However, ESBL genes *bla*_BEL_ and *bla*_CMY_ and carbapenemase genes of the *bla*_OXA-58_-like gene family, were characteristic of HWW from Burkina Faso (Table 2; Table S1C). Instead, different aminoglycoside and lincosamide resistance genes, such as *lnu*(F) and *lnu*(C), were characteristic of HWW from Benin (Table 2; Table S1C).

As a further notion, those *bla*_OXA_ variants that were significantly differentially abundant in HWW from Finland compared to Benin and Burkina Faso were predominantly those that are intrinsically carried by some specific species and encode carbapenemases (e.g., *bla*_OXA-211_-like genes) (Table 3). Instead, the *bla*_OXA_ variants characteristic for HWW from Benin and Burkina Faso were those that are typically acquired and, furthermore, do not encode carbapenemase activity (e.g., *bla*_OXA-5_ and *bla*_OXA-10_) (Table 3). For example, *bla*_OXA-5_-like genes, significantly differentially abundant in HWW from Benin, are typically carried by class 1 integrons (38).

**TABLE 3:** Differentially abundant *bla*_OXA_ variants in HWW from Benin, Burkina Faso, and Finland^*a*^

Comparison for ALDEx2	Cluster name in BLDB	Acquired/intrinsic^*b*^	Host species if intrinsic	Carbapenemase activity
Benin vs Finland				
Benin	*bla*_OXA-5_-like	A		
	*bla*_OXA-10_-like	A		
	*bla*_OXA-2_-like	A		
	*bla*_OXA-347_	A and I	*Bacteroides* spp.	
	*bla*_OXA-229_	I	Acinetobacter bereziniae	
	*bla*_OXA-46_-like	A		
	*bla*_OXA-1_-like	A		
	*bla*_OXA-209_-like	A		
Finland	*bla*_OXA-296_-like	I	Acinetobacter bohemicus	
	*bla*_OXA-58_-like	I	Acinetobacter baumannii	Yes
	*bla*_OXA-211_-like	I	Acinetobacter johnsonii	Yes
	*bla*_OXA-299_-like	I	Acinetobacter bouvetii	
	*bla*_OXA-427_-like	A		Yes
Burkina Faso vs Finland				
Burkina Faso	*bla*_OXA-10_-like	A		
	*bla*_OXA-46_-like	A		
	*bla*_OXA-2_-like	A		
Finland	*bla*_OXA-296_-like	I	Acinetobacter bohemicus	
	*bla*_OXA-211_-like	I	Acinetobacter johnsonii	Yes
	*bla*_OXA-299_-like	I	Acinetobacter bouvetii	
Benin vs Burkina Faso				
Benin	*bla*_OXA-5_-like	A		
	*bla*_OXA-10_-like	A		
	*bla*_OXA-347_	A and I	*Bacteroides* spp.	
	*bla*_OXA-2_-like	A		
	*bla*_OXA-209_-like	A		
	*bla*_OXA-46_-like	A		
	*bla*_OXA-1_-like	A		
Burkina Faso	*bla*_OXA-10_-like	A		
	*bla*_OXA-58_-like	I	Acinetobacter baumannii	Yes

a The ARGs significantly differentially abundant in each country are denoted by country. The data concerning the origin of the *bla*_OXA_ gene, whether it is intrinsic or acquired, was retrieved from Beta Lactamase Database (BLDB) (46). However, we used the expression “intrinsic” instead of “natural” due to the misleading connotation of the word “natural” in the context of antibiotic resistance.

b A, acquired; I, intrinsic.

### Carbapenemase genes.

**FIG 4:**
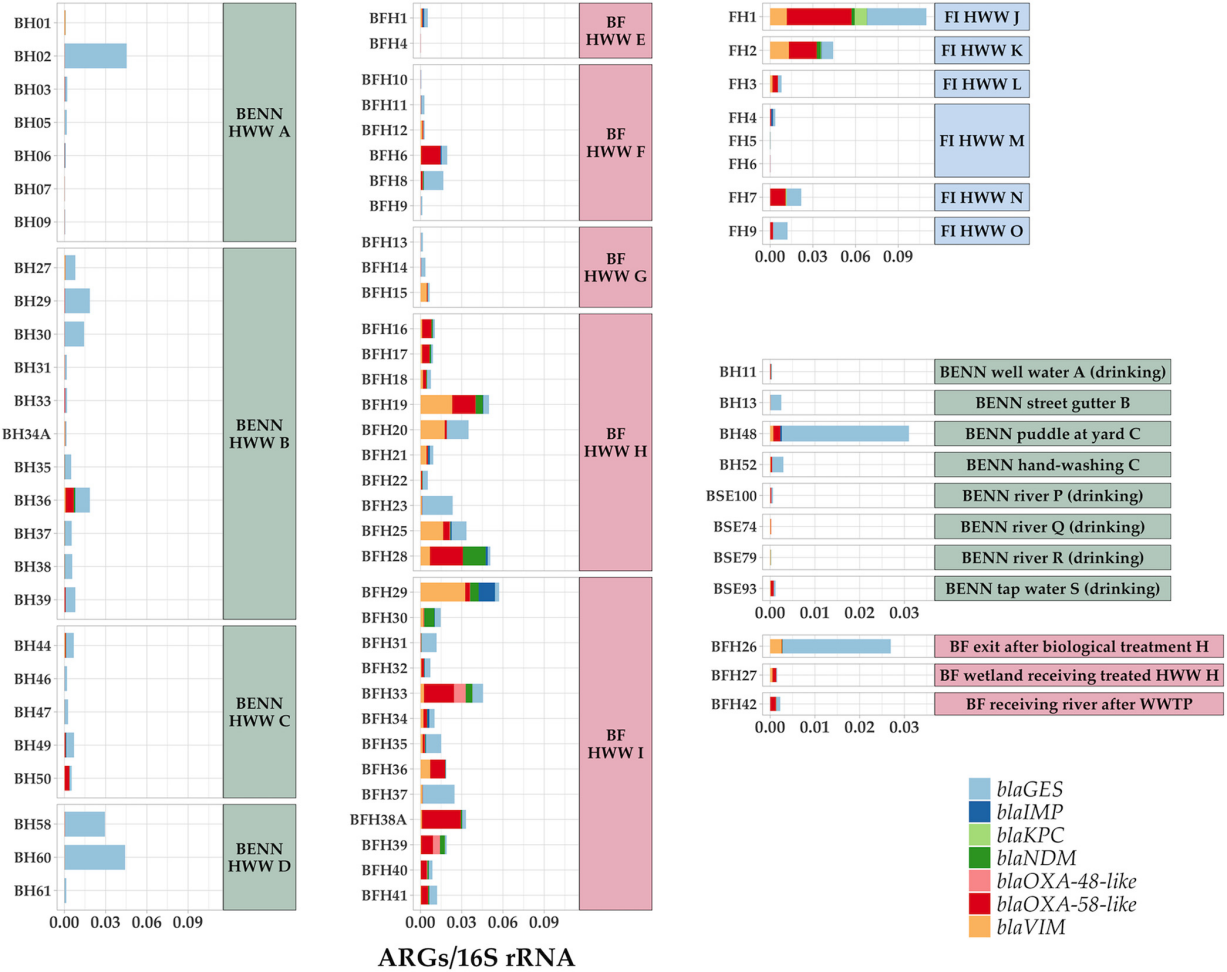
Relative abundances of carbapenemase genes in relation to 16S rRNA gene counts in HWW from Benin (left, green background), Burkina Faso (center, red background), and Finland (upper right, blue background), as well as various other water sources in Benin (center right, green background) and Burkina Faso (bottom right, red background). Note the differences in the *y* axis scales between the figures for HWW and non-HWW samples. Only variants known to encode carbapenemases were screened (46) (Table S3A).

The presence of seven acquired carbapenemase genes (*bla*_GES_, *bla*_IMP_, *bla*_KPC_, *bla*_NDM_, *bla*_OXA-48_, *bla*_OXA-58_, and *bla*_VIM_) was analyzed in detail. These ARGs were selected as their putative resistance phenotype is often associated with complex infections with minimal treatment options (16, 19, 39), especially in LMICs (2, 25). The highest relative abundance of these carbapenemase genes was observed in Finnish hospital J (Fig. 4). On the other hand, hospital M in Finland was nearly free from these carbapenemase genes (Fig. 4). In HWW from Benin, the carbapenemase genes in the *bla*_GES_ gene family seemed to dominate over other carbapenemase genes in all four hospitals (Fig. 4). *bla*_GES_ genes were also detected in the water puddle surrounding the surgery room septic tank at a Beninese hospital yard (hospital C) (Fig. 4; Fig. S2E in the Supplemental Data Repository) as well as in the water intended for hand washing in the same hospital (Fig. 4; Fig. S2F in the Supplemental Data Repository). Also, the street gutter, located ~100 m away from another Beninese hospital, was contaminated by *bla*_GES_ carbapenemase genes (Fig. 4).

**FIG 5:**
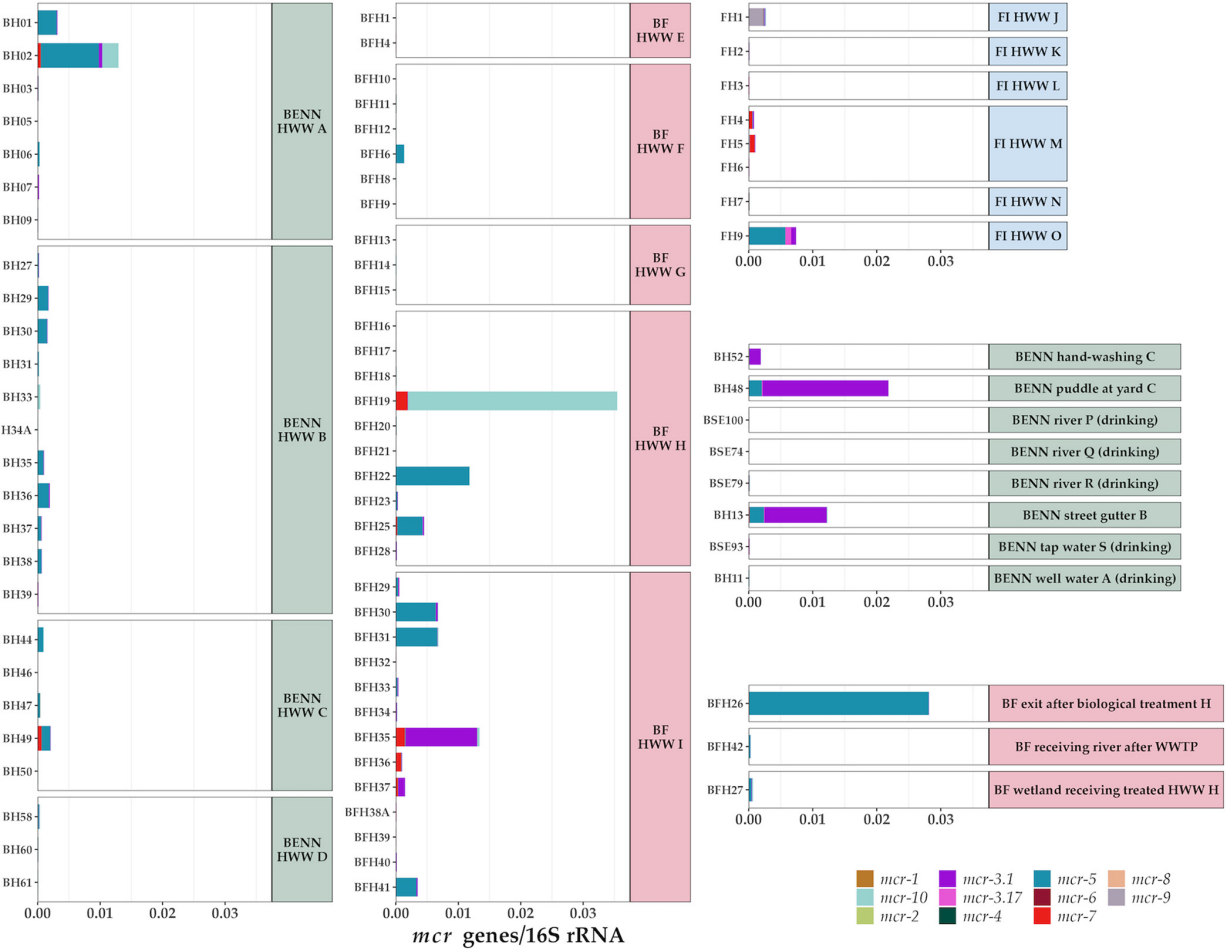
Relative abundances of *mcr* genes in relation to 16S rRNA gene counts in HWW from Benin (left, green background), Burkina Faso (center, red background), and Finland (upper right, blue background), as well as various other water sources in Benin (center right, green background) and Burkina Faso (bottom right, red background). Variants were clustered based on 90% similarity in their sequence identity using CD-HIT (80) (Table S3B).

10.1128/msphere.00538-22.3 TABLE S3 Part A lists the carbapenemase variants screened for Fig. 4 using the ResFinder database (73). Similarly, *mcr* gene variants screened for Fig. 5 are shown in part B. The table columns refer to each gene family and the rows to gene variants included in these families. Download Table S3, XLSX file, 0.01 MB. Copyright © 2023 Markkanen et al. 2023 Markkanen et al. https://creativecommons.org/licenses/by/4.0/ This content is distributed under the terms of the Creative Commons Attribution 4.0 International license.

In contrast to the homogeneity of carbapenemase genes in HWW from Benin, most of the other carbapenemase genes were present in HWW from Burkina Faso and Finland at various prevalence levels (Fig. 4). *bla*_IMP_, *bla*_NDM_, and *bla*_VIM_ were mainly detected in Burkinabe and Finnish HWW samples, and the latter was significantly differentially abundant in HWW from Burkina Faso (Fig. 4; Table S2B). *bla*_OXA-48_ was detected in two HWW samples from Burkina Faso and not at all or in very low relative abundances in samples from elsewhere (Fig. 4). Instead, *bla*_OXA-58_-like genes were present in the majority of Burkinabe and Finnish HWW samples but only in a few Beninese samples (Fig. 4). The detection of *bla*_KPC_ genes was restricted to one Finnish HWW sample (Fig. 4). The samples collected from natural waters and other drinking waters showed only low relative abundances of these carbapenemase genes (Fig. 4). However, some *bla*_OXA-58_-like were detected in tap water used for drinking in Benin (Fig. 4).

10.1128/msphere.00538-22.2 TABLE S2 Significantly differentially abundant ARGs in HWW in pairwise comparisons. ALDEx2 (36) was used to produce the differentially abundant ARGs between HWW from Benin and Finland (A), Burkina Faso and Finland (B), and Benin and Burkina Faso (C). The table columns are statistical metrics describing the significance of the differentially abundant genes in each HWW calculated by ALDEx2 (36). The Wilcoxon rank sum test, where the *P* values were adjusted for multiple testing using the Benjamini-Hochberg algorithm, is denoted as “wi.eBH.” The difference-between values for the compared groups are shown as “diff.btw,” while “diff.win” refers to within-group difference values. The “effect” value indicates the median effect size calculated by dividing the difference between by the maximum difference within all instances. The proportion of the effect size metric that overlaps zero is indicated as the “overlap” metric (36). Download Table S2, XLSX file, 0.1 MB. Copyright © 2023 Markkanen et al. 2023 Markkanen et al. https://creativecommons.org/licenses/by/4.0/ This content is distributed under the terms of the Creative Commons Attribution 4.0 International license.

In Burkina Faso, *bla*_GES_ carbapenemase genes were detected in HWW, which had gone through the biological treatment in similar relative abundances as in some of the samples of untreated HWW from the same hospital (hospital H) (Fig. 4 [note the differences in the plot scales]). Also, *bla*_VIM_ was found both in the untreated and treated water of that hospital (Fig. 4). Lower relative abundances of the studied carbapenemase genes were observed in wetlands and rivers receiving treated HWW in Burkina Faso (Fig. 4). However, the spectrum of these carbapenemase genes, namely, *bla*_OXA-58_-like, *bla*_VIM_, and *bla*_GES_, reflected the ones detected in the HWW in Burkina Faso (Fig. 4).

### Mobile colistin resistance (*mcr*) genes.

*mcr* genes were detected in several HWW samples in Benin, Burkina Faso, and Finland (Fig. 5). *mcr-5* (variants *mcr-5.1* and *mcr-5.2*) (Table S3B) was the most common of the *mcr* genes as they were found in HWW from all except two hospitals in Burkina Faso (hospitals E and G) and two hospitals in Finland (hospitals K and L) among the three countries (Fig. 5). In the few samples selected for metagenomic assembly, this gene was found to be located within a Tn*3*-like element (Fig. S7 in the Supplemental Data Repository).

In addition to the HWW samples, high relative abundances of *mcr-5* genes were also detected in the immediate and more distant surroundings of the hospitals in Benin and Burkina Faso. A very high relative abundance (2.80 × 10^−2^) of *mcr-5* was observed in the biologically treated HWW from hospital H (Fig. 5). In the wetland receiving treated HWW from the same hospital, the relative abundance was lower (4.30 × 10^−4^) but still high, considering that the sample represented a larger body of natural water. In Benin, *mcr-5* was detected in river water in a distant village and a street gutter near a hospital (hospital B) (Fig. 5). Furthermore, for hospital C, the relative abundance of *mcr-5* was greater in a puddle near the septic tanks in the hospital yard (1.99 × 10^−3^) than the average in the actual HWW of that hospital (5.24 × 10^−4^) (Fig. 5).

The next most prevalent *mcr* genes were *mcr-3.1*, *mcr-10*, and *mcr-7*, and similarly to *mcr-5*, they were also present in non-HWW water samples, such as the water intended for hand washing in the Beninese hospital C (Fig. 5). The gene *mcr-2* was not detected at all, and the lowest average relative abundance was detected for the gene *mcr-1* (Fig. 5).

### Taxonomical compositions.

The taxonomical compositions of bacteria present in septic tanks and sumps in Benin and Burkina Faso differed from those found in hospital sewers in Finland. Genera belonging to the *Bacteroidales* family showed significantly different abundances in HWW from different countries and were characteristic of the HWW from Benin and Burkina Faso (Fig. S8 and S9 in the Supplemental Data Repository). Other significantly differentially abundant taxa in HWW from Benin compared to Finland included *Chloracidobacterium*, *Geobacter*, *Aminomonas*, *Flexilinea*, *Desulfovibrio*, and genera of *Synergistetes* (Table S4A; Fig. S8A in the Supplemental Data Repository). The first two mentioned were characteristic also of the HWW from Burkina Faso (Table S4B; Fig. S8B at Supplemental Data Repository). In addition, in many HWW samples from Burkina Faso, the relative abundances of Pseudomonas and Acinetobacter were high (Fig. S9 in the Supplemental Data Repository), and the difference in their abundance in HWW from Burkina Faso versus Benin was significant (Table S4C; Fig. S8C in the Supplemental Data Repository).

10.1128/msphere.00538-22.4 TABLE S4 Significantly differentially abundant genera in HWW in pairwise comparisons. ALDEx2 (36) was used to produce the differentially abundant taxa between HWW from Benin and Finland (A), Burkina Faso and Finland (B), and Benin and Burkina Faso (C). The columns are the same as described for Table S2. Download Table S4, XLSX file, 0.02 MB. Copyright © 2023 Markkanen et al. 2023 Markkanen et al. https://creativecommons.org/licenses/by/4.0/ This content is distributed under the terms of the Creative Commons Attribution 4.0 International license.

*Coprococcus*, *Enterococcus*, *Lactococcus*, Streptococcus, *Trichococcus*, *Tessaracoccus*, *Delftia*, *Raultella*, and genera of the *Proteobacteria* family, were all significantly differentially abundant in HWW from Finland in comparison to Benin and Burkina Faso (Table S3A and B; Fig. S8A and S8B in the Supplemental Data Repository). Also, the genera that were significantly differentially abundant for HWW in Finland and not Benin were more commonly from the phylum *Pseudomonadota* (synonym *Proteobacteria*), which are typically considered the major contributors to the spread of ARGs carried by plasmids, integrons, or other MGEs (8, 40) (Fig. S8 in the Supplemental Data Repository).

A great variation was seen in the top 11 taxa in the non-HWW samples (Fig. S9 in the Supplemental Data Repository). The biologically treated HWW from Burkina Faso showed high relative abundances of *Aeromonas* and Pseudomonas. In contrast, high relative abundances of *Bacteroides* and Klebsiella were detected in the street gutter water located near hospital B (Fig. S9 in the Supplemental Data Repository). In some samples, the top 11 taxa were present only in low relative abundances. In contrast, the single genus *Polynucleobacter* dominated over other genera in a few sampled natural waters receiving treated wastewater in both Benin and Burkina Faso (Fig. S9 in the Supplemental Data Repository). The relative abundance of Acinetobacter in tap water used for drinking in Benin reached a similar level to that in many HWW samples (Fig. S9) in the Supplemental Data Repository, which possibly explained the finding of *bla*_OXA-58_-like genes in this sample mentioned earlier (Fig. 4).

## DISCUSSION

We characterized the bacterial community composition, resistome, and mobilome of 60 HWW and 11 other water samples from Benin and Burkina Faso and compared them to 8 HWW samples from Finland. Due to the lack of systematic AMR surveillance in these West African countries, available data on bacterial resistance are patchy and heterogeneous (1, 2, 22, 26, 29). Thus, the magnitude of the resistance problem and the specific ARG reservoirs are yet to be unraveled. This is the first study investigating hospital wastewater from Benin and Burkina Faso using a shotgun metagenomic approach.

Interestingly, the ARGs observed in HWW from Benin were the highest in number but probably less clinically important (16, 19, 39) than those in the HWW from Burkina Faso and Finland. While carbapenemase genes *bla*_GES_, *bla*_IMP_, *bla*_NDM_, *bla*_OXA-48_-like, *bla*_OXA-58_-like, and *bla*_VIM_ were detected at various abundances in at least one sample in HWW from Burkina Faso and Finland, *bla*_GES_ was the predominant carbapenemase in HWW from Benin. *bla*_GES_ genes were also present in the water puddle at the Beninese hospital’s yard and in water intended for visitors’ hand washing in the hospital. Thus, one route of transmission of these ARGs within hospitals might be via hands contaminated by hand washing water, which might explain the high prevalence of these genes in hospitals. Jacobs and colleagues have also drawn attention to the potentially inferior microbiological water quality in similar hand washing water tanks, which are very common in West Africa (2).

We speculate that the dominance of *bla*_GES_ carbapenemase genes might have been caused by selection pressure due to the presence of antibiotic residues in the HWW. However, as carbapenem antibiotics are used less in West African countries than in North America and Southern and Central Europe, due to their high price (15), we suggest that compounds from other antibiotic classes could have driven the selection. In fact, *bla*_GES_ genes are typically carried by class 1 integrons, in which they may be coupled with multiple other ARGs (41). Thus, we speculate that class 1 integrons (12) and coselection phenomena (25, 42) have a role in the dominance of *bla*_GES_ in HWW in Benin. That is, in the example of metagenome-assembled contigs in which quinolone ARGs (*qnrVC*) and *bla*_GES_ genes seemed to be located near each other, putatively carried by the class 1 integron gene cassette, the selection pressure targeted to the quinolone ARG would enrich both genes even in the absence of the target substrate for *bla*_GES_. However, there is a high sequence similarity among *bla*_GES_ variants, which include those conferring ESBL and carbapenem resistance. Therefore, we acknowledge the possibility that the putative lack of specificity related to the read mapping might have resulted in some misidentifications between the carbapenemase- and ESBL-encoding variants of *bla*_GES_ genes. Nonetheless, ESBL-producing bacteria are very common in African countries (43) and cause major challenges for infection control (28) as carbapenem antibiotics are less available.

Although the lowest sum of the relative abundance of ARGs was detected in HWW from Finland, some of the seven carbapenemase genes showed higher relative abundances in Finland than in HWW of the surveyed West African hospitals. For the occurrence of *bla*_OXA_ genes, the abundances of specific species present in the different HWW collection systems might have played a role, as *bla*_OXA_ variants that encode carbapenemases and are typically intrinsically carried by certain Acinetobacter species (44, 45) were characteristic of Finnish HWW. Instead, those *bla*_OXA_ variants that are typically acquired and mainly encode more-narrow-spectrum β-lactamases were significantly differentially abundant in HWW from Benin and also Burkina Faso (46), although the occurrence of the seven carbapenemase genes was not homogenous among the Finnish samples. For example, *bla*_KPC_ was limited to a single HWW sample from Finland (hospital J) and no *bla*_KPC_ gene was found from Benin or Burkina Faso. This finding aligns with local and global reports and systematic reviews (47–50), indicating that *bla*_KPC_ carbapenemase genes are spreading more profusely in Europe and North America than in Africa. Moreover, the two hospitals J and K, both part of the University Central Hospital of Helsinki, the capital of Finland, showed the highest sum of relative abundance of the seven carbapenemase genes among all HWW samples in this study. These results are somewhat surprising as, to date, Finland is known as one of the countries with the lowest level of bacterial resistance in Europe and globally (51), and the carbapenemase-producing strains detected in Finnish hospitals are relatively rare and typically associated with international travel or hospitalization (52).

One factor likely explaining our findings of the distinctive features in the HWW resistomes in Benin and Burkina Faso compared to Finland was the differences in the wastewater collection systems between the studied countries. In Finland, the hospital toilet waters containing human fecal material are directed to HWW, while for the majority of the Burkinabe and especially Beninese HWW studied here, this was not the case. Additionally, in septic tanks (Benin and Burkina Faso), the water remains stagnant, possibly giving rise to anaerobes, unlike the Finnish HWW, which flows through the system. Significant prevalence of anaerobic genera, such as *Geobacter*, *Aminomonas*, *Flexilinea*, and *Desulfovibrio*, as well as genera of *Synergistetes* and *Bacteroidales* observed in HWW from Benin and Burkina Faso and not in Finland, is in line with this speculation except for the last-mentioned taxa, which are typical human gut commensals (53). These findings also align with the previous reports on bacterial genera typically found in soil and aquatic environments (54) and previously described to dominate HWW from Benin and Burkina Faso (55). Genera of the facultatively anaerobic bacteria, such as the lactic acid bacterium *Aeromonas*, and of the anaerobic bacterium *Bifidobacterium* (56), which are considered typical human gut microbes (53), were significantly differentially abundant in the Finnish HWW instead of HWW of Benin or Burkina Faso.

Compared to many high-income countries, such as those in Northern Europe, antibiotic usage in agricultural (4, 5) and clinical (2) settings differs (15) and is less controlled—or even unregulated—in many African countries. For example, while banned in many other countries, the use of colistin as a feed additive is allowed in many LMICs (57), including in Africa (5). *mcr-5* was the most commonly detected *mcr* gene in the HWW in our study, contrary to previous reports on the prevalence of *mcr* genes globally (21) and in Africa (22, 58). This difference may be due to the methodologies used to screen for colistin resistance. Other than the best-known *mcr* genes (*mcr-1*, *-2*, and *-3*), other *mcr* gene variants are rarely targeted when screening for *mcr* genes using conventional PCR (59). Therefore, our study shows that to obtain a more realistic view of *mcr* genes in Africa, screening should be conducted for a broader set of different *mcr* genes, as was recently done by Ngbede and colleagues (60).

The *mcr-5* gene detected was embedded in a Tn*3*-like element, similar to previous reports for Salmonella enterica (61, 62) and Escherichia coli (63) plasmids and the chromosome of Cupriavidus gilardii (61). These Tn*3*-like elements are flanked by inverted repeats, which enable translocation and putatively a broad host range for the *mcr-5*-harboring element (61–63). Based on our results, the occurrences of *mcr-5* and other *mcr* genes in Benin and Burkina Faso were not restricted to HWW septic tanks. These genes were also detected in water intended for the hand washing of visitors to the hospital. The high prevalence of *mcr-5* among various samples in this study raises questions about its origin and ecology: whether it is of clinical origin or intrinsically carried by some environmental bacteria. However, the highest relative abundances of *mcr-5* were observed in samples influenced by human activity, and all four rivers in the remote village in Benin were free from this gene.

In Burkina Faso, the relative abundance of *mcr-5* in HWW, even after biological treatment, was high. Although we could not confirm the association between the *mcr-5* genes detected in the treated and the hospital-associated wastewater in the Burkinabe hospital, our study suggests the inability of the currently used wastewater treatment processes in Burkina Faso to remove the *mcr* genes. Wastewater treatment systems have indeed previously been described as inadequate in Benin and Burkina Faso (64). The situation appears to be especially critical in Cotonou, Benin, where the hospitals involved in the study did not use any kind of wastewater treatment. Groundwater in the city is extracted from the shallow aquifer, which is polluted due to unauthorized waste deposits, inadequate toilets, pit latrines, and septic tanks prone to leakage and hydraulic failure (65). This enables the continuous circling of *mcr* genes and other ARGs as the inadequately treated water is released into natural waters and used for various purposes by local people. Furthermore, in Africa, untreated wastewater is commonly used for irrigation in urban agriculture, possibly enabling the dissemination of ARGs to fresh produce (66, 67). Our study shows that untreated or even treated hospital wastewater, which can leak into groundwater and the environment, may carry clinically hazardous antibiotic-resistant bacteria or resistance genes.

Based on our results from nine hospitals, we can state that clinically important ARGs are circulating in Beninese and Burkinabe hospitals and their surroundings. There were differences in the HWW collection systems in Benin and Burkina Faso compared to Finland. This, among other factors, seems to be reflected to some extent in the taxonomical compositions and, therefore, resistomes found in these HWW. However, taxonomy explained the variance in the resistomes from the different countries only partially, at least at the genus level. Although there were fewer dissimilarities in the resistomes between HWW from Benin and Burkina Faso than in the comparisons with Finland, it is important to consider the differences among West African countries in AMR surveillance.

## MATERIALS AND METHODS

### Sample description.

Hospital wastewater (HWW) samples were collected in Benin from four hospitals (hospitals A to D; *n* = 26) and in Burkina Faso from five different hospitals (hospitals E to I; *n* = 34) in November and December 2019. In Finland, HWW samples were collected from six different hospitals (hospitals J to O; *n* = 8) in January 2020. The various wards, clinics, and other units typically had their own septic tanks or sumps in the Beninese and Burkinabe hospitals. In Burkina Faso, the samples were mainly from septic tanks or sewers in the hospital area. In Benin, none of the hospitals was connected to a sewer system, and the samples were from septic tanks or sumps (unstructured wastewater wells), which were never emptied to our knowledge. In most cases, the toilet water was not directed into these sumps. For comparison, 11 non-HWW water sources were sampled. These included samples from the tap [BENN tap water S (drinking); *n* = 1] and river waters [BENN river P, Q, R (drinking); *n* = 3] used for drinking in a remote countryside village in the community of Savalou in central Benin as well as drinking water from a well located in a 100-m distance from hospital A in Benin [BENN well water A (drinking); *n* = 1]. Street gutter water near hospital B (in a 100-m distance) (BENN street gutter A; *n* = 1) and a water puddle at hospital C yard next to HWW septic tank (BENN puddle at yard C; *n* = 1) and a tank distributing water for hand washing in hospital C in Benin (BENN hand-washing C; *n* = 1) were also sampled. In Burkina Faso, samples from biologically treated HWW from hospital H (BF exit after biological treatment H; *n* = 1) and a wetland receiving this water (BF wetland receiving treated HWW H; *n* = 1) were collected. Additionally, one sample was collected from wastewater treated in a local wastewater treatment plant (WWTP) and destined for a river in Burkina Faso (BF receiving river after WWTP; *n* = 1) (Table 1). Detailed sample descriptions are provided in the Data Set S1, Sheets 1 and 2, in the supplemental material. Illustrative pictures and a map indicating sample collection regions are shown in Fig. S1 and S2 in the Supplemental Data Repository, https://data.mendeley.com/datasets/9wxb37t49z/1.

### DNA extraction and metagenomic sequencing.

Water samples were collected into 1- L bottles and transported to a laboratory on ice. They were kept at +4°C until processed within 24 h. A volume of 50 to 100 mL was filtered through a 0.2-μm-pore polycarbonate filter (Whatman; GE Healthcare Life Sciences) using a portable vacuum pump (Millivac-Mini vacuum pump XF54; Millipore, Merck). DNA was extracted from the filters using the Qiagen Dneasy PowerWater DNA kit following the manufacturer’s instructions. The concentration and quality of the extracted DNA were determined with a NanoDrop spectrophotometer. Altogether, 79 samples were subjected to shotgun metagenomic sequencing using Illumina Novaseq6000 with Nextera XT library preparation at the Institute of Biotechnology, University of Helsinki.

### Bioinformatic analyses.

All quality control and read mapping analyses were run using an in-house Snakemake (v.5.3.0) (68) workflow. Briefly, the quality control steps included in the workflow were performed using FastQC (v.0.11.8) (69) and MultiQC (v.1.9) (70), with adapter and low-quality read removal using Cutadapt (v.2.7) (71) (parameters -O 10 -m 30 -q 20). Nucleotide sequence reads were mapped using Bowtie2 (v.2.4.1) (72) (parameters -D 20 -R 3 -N 1 -L 20 -I S,1,0.50) against the ResFinder database (v.3.2; downloaded on 28 June 2020) (73). The reads were sorted and filtered using SAMtools (v.1.9) (74), such that the reads mapping as pairs or alone were calculated as a single count. Mobile genetic elements were identified by mapping the reads with Bowtie2, similarly to the procedure described above, against the MobileGeneticElementDatabase (56) (https://github.com/KatariinaParnanen/MobileGeneticElementDatabase; downloaded on 28 June 2020), consisting of 2,714 unique MGE sequences, including transposons, integrons of classes 1, 2, and 3, the integron-associated disinfectant resistance gene *qacE*Δ, and standard insertion sequences (ISs) and ISs with insertion sequence common regions (ISCRs).

Taxonomic profiling was performed using both Metaphlan3 (v.3.0.1) (75) and Metaxa2 (v.2.2.1) (76). Metaphlan3 was run to achieve both relative abundances and “absolute abundances” defined by the program developers using the parameter -t rel_ab_w_read_stats. The outputs from the former were used for ordinations and the latter for diversity analyses with vegan package (v2.6.2) (77, see below). In both cases, the merged abundance tables were modified so that only taxa that were identified to species level were included in the downstream analyses. However, knowing the limitations of species-level taxon identification using shotgun metagenomics (78), the results were presented only at the genus level after taxon agglomeration by the function tax_glom from the phyloseq package (v.1.40.0) (79) prior to analyses.

The counts for bacterial 16S rRNA from Metaxa2 were used to normalize ARGs and MGE counts to obtain relative abundances. Gene lengths were taken into account in the normalization. Three HWW samples were collected in duplicate (Data Set S1, Sheet 2). These replicates were not included in the statistical analyses but were used to evaluate the selected methods for detecting resistance. The sums of the relative abundances of ARGs in the replicated sample pairs were highly similar to each other (Fig. S10 in the Supplemental Data Repository).

All resistance genes found in the ResFinder database (73) were clustered into gene families based on 90% similarity in their sequence identity using CD-HIT (v.4.8.1) (80). Data available in the Beta-Lactamase DataBase (BLDB) (46) were used to confirm the carbapenemase activity of the variants of *bla*_OXA_ gene families as well as other carbapenemase genes studied here (Table S3A). For instance, only *bla*_GES_ variants known to encode carbapenemases were included in the visualization (Fig. 4), while those encoding ESBL phenotypes were not. Similarly, ARG clustering of 90% shared nucleotide identity was applied to group *mcr* variants (Table S3B).

To study the genetic environment of ARGs, a subset of samples was assembled into contigs with MEGAHIT (v.1.2.8) (81) with parameters –min-contig-len 1000 -m 32000000000. The anvi’o (v.7) (82) and Bandage (83) programs were applied to visualize the genetic environments (Fig. S5 and S7 in the Supplemental Data Repository). For the *mcr-5* gene, one to two *mcr-5*-positive samples from all studied countries were selected for the assembly and the analysis of the genetic background. The same samples were used to search putative integron-carried multidrug resistance gene cassettes. Those putative gene cassettes with multiple ARGs encoded by the same fragment are represented in Fig. S4 in the Supplemental Data Repository.

### Statistical analyses. (i) General.

All statistical analyses described below were performed for the 68 HWW samples (Table 1; Data Set 1, Sheet 1). The sum of the relative abundances for ARGs, MGEs, and *intI1* was obtained using 16S rRNA gene counts and gene lengths to normalize the count data. As the data did not fulfill the assumptions of normality, a Kruskal-Wallis test from the stats package (v.4.2.0) (84) was applied to study whether the differences between countries were significant. Pairwise Wilcoxon rank sum tests adjusted by Benjamini-Hochberg from the stats package (v.4.2.0) (84) were performed to determine which comparisons were significant for ARGs and *intI1*. Pearson correlations for the relative abundances of ARGs and MGEs were computed using package ggpubr (v 0.4.0.999) (85, see below).

### (ii) Compositional analyses.

Next-generation sequencing (NGS) data are compositional as they contain only relative information (37). By ignoring this compositional nature of NGS data, results conducted by traditional normalization methods may suffer from technical artifacts due to sequencing depth limitations (86). The ANOVA-Like Differential Expression tool for high-throughput sequencing data (ALDEx2) (v.1.28.0) (36) was applied to study the divergent features of the resistomes in HWW from each studied country. ALDEx2 handles the compositionality of the data by applying suitable data transformations. To study the differentially abundant ARGs in this study, additive log ratio (alr) transformation was used with the 16S rRNA gene as the denominator gene. First, the ARG count data were split, so that pairwise comparisons between countries were possible (e.g., Benin versus Finland). The command aldex.clr(df, conditions, denom = ref) excludes the features with zero count in all samples and performs the alr transformation with the selected reference gene. The significance of the comparisons was tested using the Wilcoxon rank test [command aldex.ttest(x, paired.test = FALSE, verbose = FALSE)], in which Benjamini-Hochberg corrections control false-positive identifications. Finally, the effect sizes and the within- and between-condition values were estimated with the command aldex.effect. ALDEx2 (36) was also applied to study the differentially abundant taxa between the HWWs from different countries. For that, centered log ratio transformation, which uses the geometric mean of the sample vector as the reference, was applied to Metaphlan3 count data (generated using the parameter -t rel_ab_w_read_stats), and the significance of the comparisons was tested similarly as described above.

For principal-component analysis (PCA) ordinations, clr-transformed ARG, MGE, and taxon counts (generated by Metaphlan3 with parameter -t rel_ab_w_read_stats) were visualized using microViz (v.0.9.1) (87) with the command count_data %>% tax_transform(“clr”) %>% ord_calc(method = “PCA”) %>% ord_plot(color = “country”). For PCA, taxa were fixed to the genus level as described earlier. The significance of the distances between samples from country pairs was calculated using Aitchison distance on the untransformed count data by microViz (87) [command count_data %>% dist_calc(“aitchison”) %>% dist_permanova(variables = c(“country”), n_perms = 9999, seed = 12345)].

All statistical analyses were performed in RStudio (v.4.2.0), and the results were visualized using ggplot2 (v.3.3.6) (88) and patchwork (v.1.1.1) (89). Vector maps were drawn using the packages rnaturalearth (v.0.1.0) (90), ggspatial (v.1.1.6) (91), and maps (v.3.4.0) (92).

### Ethical permission.

The ethical permissions for the project were received from Comité National d´Ethique pour la Recherche en Santé under the Health Ministry in Benin and Comité d´Ethique pour la Recherche en Santé under the Health Ministry in Burkina Faso.

### Data availability.

The data for this study have been deposited in the European Nucleotide Archive (ENA) at EMBL-EBI under accession no. PRJEB47975. They will be public upon article publication. All custom codes used for the analyses are available from https://github.com/melinamarkkanen/AMRIWA upon article publication. Analysis scripts were provided for the reviewers prior to publication.
